# Mental Health Among People Presenting for Care of Physical Symptoms: The Factors Associated with Suicidality and Symptoms of Depression and Anxiety are Similar Across Specialties

**DOI:** 10.1177/24705470231169106

**Published:** 2023-04-18

**Authors:** Sina Ramtin, Kathleen Carberry, Maria Correa, David Ring, Carol Alter, Donna Shanor

**Affiliations:** 1 Department of Surgery and Perioperative Care, Dell Medical School, University of Texas at Austin, Austin, TX, USA

**Keywords:** symptoms of depression, thoughts of suicide, self-harm, GAD, PHQ

## Abstract

**Background:**

To identify differences in thoughts of suicide and symptoms of depression and anxiety by specialty among people presenting for care of physical symptoms, we analyzed data from routine mental health measurement in a small multispecialty practice and asked: 1. Are there any differences in suicidality (analyzed as an answer of 1 or greater or 2 or greater on the Patient Health Questionnaire [PHQ] question 9) in non-specialty and various types of specialty care? 2. Are there any factors—including specialty–associated with symptoms of depression (mean PHQ score), PHQ thresholds (greater than 0, 3 or greater, 10 or greater), Generalized Anxiety Disorder instrument [GAD] score of 3 or greater, and either GAD score 3 or greater or PHQ score 3 or greater? and 3. What factors are associated with referral to a social worker?

**Methods:**

As part of routine specialty and non-specialty care, 13,211 adult patients completed a measure of symptoms of depression (PHQ) that included a question about suicidality and a measure of symptoms of anxiety (GAD). Factors associated with suicidality and symptoms of depression and anxiety at various thresholds, and visit with a social worker, were sought in multivariable models.

**Results:**

Accounting for potential confounding in multivariable analyses, a score higher than 0 on the suicidality question (present in 18% of people) was associated with men, younger age, English-speakers, and neurodegenerative specialty care. Symptoms of depression on their continuum and using various thresholds (28% of people had a PHQ score greater than 2) were associated with non-Spanish-speakers, younger age, women, and county insurance or Medicaid insurance. Care from the social worker was associated with PHQ score of 3 or greater and having any suicidal thoughts (score of 1 or greater on question 9) but was less common with Medicare or Commercial Insurance and less common in the unit treating cognitive decline.

**Conclusion:**

The notable prevalence of symptoms of depression and suicidality among people presenting for care of physical symptoms across specialties and the relatively similar factors associated with suicidality, symptoms of depression, and symptoms of anxiety at various thresholds suggests that both non-specialty and specialty clinicians can be vigilant for opportunities for improved mental health. Increased recognition that people seeking care for physical symptoms often have mental health priorities has the potential to improve comprehensive care strategies, alleviate distress, and reduce suicide.

## 1. Introduction

Both specialty and non-specialty care units increasingly measure symptoms of depression and anxiety as a routine part of whole-person, comprehensive care.^1,2^ Patients visiting our multispecialty group practice complete a measure of symptoms of depression and suicidality (Patient Health Questionnaire; PHQ) and a measure of symptoms of anxiety (Generalized Anxiety Disorder (GAD) questionnaire). On site care from social workers supported by psychiatrists is integrated into care. This is a relatively novel practice strategy, designed to identify and treat opportunities for improved mental health in a non-mental health care setting.

The first identification of an opportunity for improved mental health may occur in the care of physical symptoms.^3–8^ And many people who die by suicide had recently passed through specialty or non-specialty medical care as an outpatient or inpatient.^9,10^ Consider also that symptoms of depression on their continuum correlate notably with symptom intensity and magnitude of incapability. Furthermore, people presenting for care of physical symptoms may not be forthright on mental health measures and may complete them hastily^8,11,12^ Click or tap here to enter text. All evidence considered, it may be worthwhile to address mental health symptoms among people seeking care for physical symptoms even if they do not meet a threshold for an estimated diagnosis of major depression.

The tendency of clinicians and lay people to dichotomizing mental health and focus on diagnosis of major depression or generalized anxiety disorder is inconsistent with evidence that lesser degrees of symptoms of depression and anxiety on their continuum have notable associations with discomfort and incapability,^13^ and risks reinforcing mental health stigma.^8,11,12^ Dichotomizing mental health combined with inattentiveness to lesser degrees of feelings of distress may both reinforce mental health stigma as well as overlook opportunities for improved health and avoidance of low-value care that distracts from mental health priorities. While our unit is well-poised to identify and address mental health in specialty and non-specialty care of physical symptoms, we noticed that positive screenings for suicidality were not always noticed and acted upon and that even small amounts of depression could signal potential benefits from mental or social health care. Most experimental evidence we identified addressed the diagnostic performance characteristics of screening instruments such as the PHQ and the GAD. There was less experimental evidence regarding the degree to which clinician of various specialties can anticipate suicidality and symptoms of depression and anxiety at various among people seeking care for physical symptoms, and factors associated with utilization of a social worker when one is readily available in the office.^14–18^

We, therefore, analyzed the data from our practices and asked: 1. Are there any differences in the rate of suicidality in two grades (analyzed as an answer of 1 or greater or 2 or greater on question 9 of the PHQ) in non-specialty and various types of specialty care? 2. Are there any factors—including specialty–associated with symptoms of depression (mean PHQ score), PHQ thresholds (> 0, 3 or greater, 10 or greater), GAD score of 3 or greater, and either GAD score 3 or greater or PHQ score 3 or greater? and 3. What factors are associated with referral to a social worker when one is readily available in the office?

## 2. Methods

### Study Design and Setting

This study was approved by our Institutional Review Board (IRB). We performed a retrospective database study of all new or return adult patients (≥18 years) between January 2017 and January 2021 seeking care in one of several specialty care units at a university in a large urban area of the United States. This database includes demographics (age, gender, race, ethnicity, and language), type of insurance, specialty unit, and whether the patient saw a social worker as part of their care. Medicaid, county safety net, and self-pay insurance status reflect socioeconomic disadvantage in our setting. The surveys were distributed according to each patient's language preference. As part of routine care, patients completed a measure of symptoms of depression, the Patient Health Questionnaire (first the 2-question version and then if the score was greater than 3, a 9-question version that included question 9, which addresses suicidal thoughts and thoughts of self-harm; PHQ2/9) and a measure of symptoms of anxiety, the Generalized Anxiety Disorder (first the 2-question version and then if the score was greater than 3, a 7 question version; GAD-2/7).^19,20^

A total of 9248 women (70%) and 3963 men (30%) with an average age of 48 (standard deviation, 17) completed the questionnaires (Appendix 1). Around half of the patients visited the musculoskeletal specialty care unit (Table 1A, B, C, and Appendix 1).

**Table 1.** A. Symptoms of depression and anxiety by integrated practice unit

Unit	Number of patients	% of all patients	Number of patients seen by social worker	% patients seen by social worker	Mean PHQ	SD	Median PHQ	IQR	Number of people with PHQ > 0	% PHQ > 0	Number of people with PHQ ≥ 3	% PHQ ≥ 3	Number of people with PHQ ≥ 10	% PHQ ≥ 10
Primary Care	1633	12%	165	10%	1.8	4.2	0	0–2	589	36%	229	14%	123	8%
Medical Specialties	1222	9%	102	8%	2.7	5.2	0	0–2	520	43%	257	21%	170	14%
Comprehensive Memory	251	2%	36	14%	3.7	6.4	1	0–2	131	52%	61	24%	46	18%
Women's Health	3278	24%	428	13%	3.3	5.5	1	0–2	1707	52%	904	28%	640	20%
Multiple Sclerosis	420	3%	97	23%	4.0	6.2	1	0–5	244	58%	117	28%	82	20%
Musculoskeletal	6294	48%	886	14%	3.8	6.2	1	0–4	3168	50%	2052	33%	1453	23%
Comprehensive Pain Management	113	1%	39	35%	5.7	6.8	2	0–12	72	64%	50	44%	38	34%
TOTAL	13,211	100%	1753	13%	3.4	5.8	1	0–2	6431	49%	3670	28%	2552	19%

B. Symptoms of depression and anxiety by integrated practice unit						
Unit	Number of patients with PHQ ≥ 3 seen by social worker	% patients with PHQ ≥ 3 seen by social worker	Number of people that completed Question 9	Number of people with positive Question 9 (suicidality)	Percentage of total people seen by IPU with suicidality	Number of people with Question 9 (suicidality) > 1
Primary Care	47	21%	175	27	2%	11
Medical Specialties	41	16%	156	30	2%	13
Comprehensive Memory	8	13%	59	17	7%	11
Women's Health	172	19%	628	96	3%	25
Multiple Sclerosis & Neuroimmunology	39	33%	89	28	7%	9
Musculoskeletal	520	25%	1458	263	4%	103
Comprehensive Pain Management	18	36%	27	2	2%	1
TOTAL	845	23%	2592	463	4%	173

C. Symptoms of depression and anxiety by integrated practice unit												
Integrated Practice Unit	Percentage of patients who visit social worker with suicidality	Number of people with Question 9 (suicidality) > 1	Percentage of patients who visit social worker with question 9 > 1	Percentage completed Question 9 that had suicidality	Number of patients with suicidality seen by social worker	% of patients with suicidality seen by social worker	GAD Mean	SD	Median	IQR	Number of people with GAD > 3	% GAD > 3
Primary Care	2%	11	1%	15%	14	52%	1.90	3.81	0	0–2	281	17
Medical Specialties	2%	13	1%	19%	4	13%	2.22	4.25	0	0–2	249	20
Comprehensive Memory	7%	11	4%	29%	1	6%	3.59	5.73	1	0–2	63	25
Women's Health	3%	25	1%	15%	32	33%	3.33	4.94	1	0–4	940	29
Multiple Sclerosis & Neuroimmunology	7%	9	2%	31%	12	43%	3.61	5.40	1	0–4	120	29
Musculoskeletal	4%	103	2%	18%	118	45%	2.75	4.99	0	0–2	1641	26
Comprehensive Pain Management	2%	1	1%	7%	1	50%	4.55	6.17	2	0–6	49	43
TOTAL	4%	173	1%	18%	182	39%	2.80	4.85	1	0–2	3343	25

PHQ: Patient Health Questionnaire; GAD: Generalized Anxiety Disorder; SD: Standard Deviation; IQR: Interquartile Range; IPU: Integrated Practice Units

### Measurements

The PHQ-2 contains the first 2 questions of the PHQ-9 and asks about the frequency of depressed mood over the past 2 weeks. Response options range from 0 (not at all) to 3 (nearly every day), with a maximum of 6 points. A higher score indicates greater symptoms of depression. Patients with scores of 3 or higher on the PHQ-2 were asked to complete the 9-item version (PHQ-9).

The Generalized Anxiety Disorder-2 is the first 2 questions of the GAD-7, a 7-item measure of symptoms of anxiety over the past 2 weeks. The total score ranges from 0 to 21, and higher scores indicate greater symptoms of anxiety. Patients with scores of 3 or higher on the GAD-2 were asked to complete the 7-item version (GAD-7).

### Statistical Analysis

To identify factors associated with 2 thresholds for suicidality on item 9 (score of 1 or greater and score of 2 or greater), the PHQ Score, various PHQ score thresholds (greater than 0, 3 or greater, 10 or greater), GAD score of 3 or greater, and either PHQ score 3 or greater or GAD score of 3 or greater, and whether or not the patient was seen by a social worker, we first performed bivariate analyses (Appendix 2). While this is not necessary for a large dataset, and did not address our study questions, we were interested in the descriptive statistics that would be provided by bivariate analysis. Mann–Whitney U tests evaluated the relationship between categorical variables and non-parametric continuous variables, Kruskal–Wallis H tests the association between non-parametric continuous variables and categorical variables with more than two groups, and Spearman rank correlations for comparison of continuous variables. All variables with *P *< .10 were moved to multivariable regression analysis. We used logistic regression to identify factors associated with various thresholds of suicidality (Table 2). For non-parametric discrete response variables with a relative floor effect such as PHQ and GAD scores, we used negative binomial regression analysis. All *P*-values below 0.05 were considered statistically significant (Table 3).

**Table 2.** Logistic regression analysis of patient factors associated with the suicidality (PHQ 9 > 0).

Variables	Odd's ratio (95% Confidence Interval)	Standard Error	*P*-value
Gender			
Woman	*reference value*		
Man	1.11 (0.88 to 1.41)	0.134	.37
Department			
Primary Care	*reference value*		
Medical Specialties	1.39 (0.78 to 2.48)	0.410	.26
Comprehensive Memory Center	2.69 (1.31 to 5.51)	0.985	**.01**
Women's Health	1.08 (0.67 to 1.73)	0.261	.76
Multiple Sclerosis & Neuroimmunology	2.55 (1.39 to 4.69)	0.793	**.003**
Musculoskeletal	1.36 (0.88 to 2.11)	0.305	.17
Comprehensive Pain Management	0.46 (0.10 to 2.07)	0.354	.31
Language			
Spanish	*reference value*		
English	1.60 (1.17 to 2.19)	0.256	**.003**
Other	1.84 (0.84 to 4.05)	0.740	.13
Age	0.991 (0.984 to 0.999)	0.004	**.02**

Bold indicates statistical significance, *P *< .05. Race and ethnicity were dropped because of the collinearity with language. PHQ-9= Patient Health Questionnaire, 9-item. GAD = General Anxiety Disorders.

**Table 3.** Negative binomial regression analysis of patient factors associated with PHQ score.

Variables	Regression Coefficient (95% Confidence Interval)	Standard Error	*P-value*
Gender			
Woman	*reference value*		
Man	−0.15 (−0.23 to −0.07)	0.041	**<**.**001**
Department			
Primary Care	*reference value*		
Medical Specialties	0.29 (0.14 to 0.44)	0.075	**<**.**001**
Comprehensive Memory Center	0.52 (0.25 to 0.79)	0.138	**<**.**001**
Women's Health	0.42 (0.30 to 0.54)	0.063	**<**.**001**
Multiple Sclerosis & Neuroimmunology	0.76 (0.55 to 0.97)	0.106	**<**.**001**
Musculoskeletal	0.43 (0.32 to 0.55)	0.059	**<**.**001**
Comprehensive Pain Management	0.76 (0.39 to 1.14)	0.194	**<**.**001**
Language			
Spanish	*reference value*		
English	0.40 (0.29 to 0.51)	0.056	**<**.**001**
Other	0.32 (0.01 to 0.63)	0.158	.04
Insurance status			
County insurance	*reference value*		
Medicaid	0.08 (−0.10 to 0.25)	0.089	.386
Medicare	−0.44 (−0.56 to −0.33)	0.095	**<**.**001**
Commercial	−0.79 (−0.89 to −0.69)	0.060	**<**.**001**
Self-pay	−0.51 (−0.70 to −0.33)	0.093	**<**.**001**

Bold indicates statistical significance, *P* < .05. Race and ethnicity were dropped because of the collinearity with language. PHQ-9= Patient Health Questionnaire.

## 3. Results

### Factors Associated with Thresholds of Suicidality

Accounting for potential confounding including gender, specialty, language, and age in multivariable analysis, a score higher than 0 on the suicidality question was associated with men, younger age, English speakers, and neurodegenerative specialty care (Appendix 2, Table 2). Accounting for potential confounding including gender, specialty, Ethnicity, and insurance status in multivariable analysis, a score higher than 1 on the suicidality question was associated with women's health specialty care, comprehensive memory center, Medicare, and commercial insurances (Appendices 2 and 3).

Four-hundred and sixty-three of 2592 patients (18%) who had a PHQ-2 score greater than 2 and completed question number nine on the PHQ-9 had some level of suicidality.

### Factors Associated with Symptoms of Depression on Their continuum

Accounting for potential confounding including gender, specialty, language, and insurance status in multivariable analysis, greater symptoms of depression on their continuum were associated with women, non-Spanish speakers, county or Medicaid insurance, and specialties other than primary care (Table 3).

### Factors Associated with Various Threshold Scores on the PHQ and GAD Measures

In total, about half (6431 of 13,211; 49%) of the patient had some symptoms of depression. Accounting for potential confounding in multivariable analysis, PHQ score greater than zero, 3 or greater, and 10 or greater, GAD score 3 or greater, either PHQ of 3 or greater or GAD of 3 or greater, and GAD score on the continuum were all associated with non-Spanish-speakers, younger age, women, county or Medicaid insurance, and specialty care (Appendices 4-9), except that age and gender, were not associated with a PHQ score of 3 or greater, and age was not associated with a PHQ score of 10 or greater.

### Factors Associated with Care from a Social Worker

Accounting for potential confounding including gender, specialty, language, insurance status, PHQ threshold of 3, and suicidality in multivariable analysis, receiving care from the in-office social worker was associated with PHQ score of 3 or greater and having any suicidal thoughts (score of 1 or greater on question 9) but was less common with Medicare or Commercial Insurance and less common in the unit treating cognitive decline (Table 4). One-hundred and eighty-two of 463 patients with suicidality (39%) were evaluated by a social worker.

**Table 4.** Logistic regression analysis of patient factors associated with the seen by social workers.

Variables	Odd's ratio (95% Confidence Interval)	Standard Error	*P*-value
Gender			
Woman	*reference value*		
Man	0.81 (0.66 to 1.01)	0.090	.064
Department			
Primary Care	*reference value*		
Medical Specialties	0.60 (0.34 to 1.06)	0.174	.076
Comprehensive Memory Center	0.38 (0.16 to 0.95)	0.177	.**038**
Women's Health	0.76 (0.49 to 1.17)	0.166	.207
Multiple Sclerosis & Neuroimmunology	1.63 (0.91 to 2.93)	0.487	.1
Musculoskeletal	0.96 (0.64 to 1.45)	0.202	.856
Comprehensive Pain Management	1.27 (0.51 to 3.17)	0.593	.61
Language			
Spanish	*reference value*		
English	1.17 (0.91 to 1.51)	0.153	.228
Other	0.51 (0.23 to 1.12)	0.204	.093
Insurance status			
County insurance	*reference value*		
Medicaid	1.08 (0.75 to 1.57)	0.204	.669
Medicare	0.65 (0.50 to 0.85)	0.087	.**001**
Commercial	0.65 (0.48 to 0.89)	0.101	.**006**
Self-pay	0.70 (0.41 to 1.18)	0.186	.178
PHQ			
<3	Reference value		**<**.**001**
>=3	5.80 (2.34 to 14.39)	2.688	
Suicidality			
No	Reference value		**<**.**001**
Yes	2.11 (1.70 to 2.63)	0.236	

Bold indicates statistical significance, *P *< .05. Race and ethnicity were dropped because of the collinearity with language. GAD was dropped because of the collinearity with PHQ. GAD = General Anxiety Disorder.

## 4. Discussion

Our unit is designed to address mental health in specialty and non-specialty care, and we identified areas in need of better data to drive improvement. In particular, we sought a better understanding of factors associated with suicidality, symptoms of anxiety and depression, and with seeing a social worker who is integrated into care. Our goal was to identify opportunities to improve our screening and management strategies for identifying and addressing mental health and social health concerns. In a study of adult patients who presented for specialty or non-specialty care for the care of physical symptoms, all of whom completed mental health screening as part of routine care, we found that opportunities for improved mental health were common and associated with non-Spanish-speakers, younger age, women, lower socioeconomic status (safety net medical insurance), and specialty care.

### Limitations

The study should be considered in light of its shortcomings. First, this was a relatively small amount of data at a single institution with limited specialty representation and relatively high numbers of socioeconomically disadvantaged patients in the musculoskeletal and women's health units. The observation of differences by specialty and many of the associations (which depend on variation within the sample, rather than absolute rates) are likely reproducible in other samples. The absolute rates may not generalize. Second, periodic technical issues led to missing questionnaires, but this occurred at random and should not influence the analysis. Third, because some health care units such as gastroenterology, infectious disease, dermatology, and oncology (medical specialties), had a limited number of patients, we were unable to evaluate each of them separately. Fourth, multiple testing might seem to be an issue, but the reader should focus on the MV analysis for the primary study question as the key analysis with all other analyses being secondary and hypothesis generating. Fifth, the bivariate analysis was performed largely for descriptive statistics, but the primary analysis for each study question was a single multivariable analysis. Sixth, this is a database study, so the details of the medical status or level of functional impairment were not readily available. Seventh, the database does not provide information on the number of patients who did not receive social work evaluation due to already being connected to psychosocial support. Eighth, the database does not capture the number of patients who may have benefited from social work services based on factors other than questionnaire scores, and the number of patients who might benefit from social work assistance who were already receiving adequate assistance.

### Factors Associated with Suicidality

The observation that a score higher than 0 on the suicidality question was associated with men, younger age, English speakers, and neurodegenerative disorders can inform comprehensive care strategies. A score of greater than zero on PHQ question 9 alone may not be a suitable trigger for mental health referral, but there are likely many benefits of clinician review of these measures and discussion of the responses with patients.^14,21^ A threshold for a moderate-to-strong association in logistic regression is an odds ratio (OR) of 2 or higher. Given that the majority of the ORs included in this analysis met or exceeded this threshold, it can be concluded that there is a moderate to strong association between the variables under investigation.^22^ It is clear from these and other data that people who enter care specific to physical symptoms and pathophysiology often have important feelings of worry, despair, and suicidality that non-mental health clinicians can anticipate and be prepared to address, even if they don’t meet specific thresholds.^13,23^ These data remind us that many people seeking care for physical symptoms can benefit from attention to their mental and social health. A combination of screening questionnaires, discussion of those questionnaires, and further assessment when appropriate to determine patients’ needs from available integrated mental health represents a comprehensive, whole-person approach to health that, evidence suggests, is more effective than addressing pathophysiology alone.^7,8,24–27^

### Factors Associated with Symptoms of Depression and Anxiety

The finding that greater symptoms of depression are associated with specialties other than primary care—with nearly 1 in 3 people presenting for specialty care having a PHQ score of 3 or greater, and about 1 in 25 reporting some suicidality in our setting—reinforces the importance of 1) non-specialists considering potential mental health support when considering specialty referral, 2) coordination of mental and physical health care between non-specialty and specialty care, 3) anticipation by specialists that a notable percentage of their patients will have an illness influenced in important ways by mental and social factors, and 4) training specialist and non-specialists in strategies for both routinely reviewing questionnaires and considering mental health as well as making mental and social health comfortable topics of conversation. The observation that the various thresholds for symptoms of depression or anxiety were associated with relatively similar factors, most associated with socioeconomic disadvantage,^28,29^ combined with the knowledge that people don’t always complete mental health questionnaires forthrightly,^30–32^ suggests that any degree of symptoms of depression can spark a helpful discussion that ensures no opportunities for improved mental health are overlooked. The finding that men, older age, and better socioeconomic status (non-safety net insurance) are protective is consistent with a prior study in adults selected to represent major demographic groups in the US that younger age, women, Hispanic ethnicity, and people of color had greater symptoms of depression.^33^ The coefficient of an explanatory variable in a negative binomial regression represents the change in the log of the expected count of the response variable associated with a one-unit change in the explanatory variable, holding all other variables constant. In our setting, speaking Spanish (distinct from Hispanic ethnicity) was associated with fewer symptoms of depression, which may reflect cultural factors associated with mental health or how mental health questionnaires are completed. We consider mental health questionnaires a potentially helpful, but not sufficient method for being comprehensive in our care and addressing all opportunities for improved mental health.

### Factors Associated with Care from a Social Worker

The observation that a visit with a social worker integrated into care is associated with a higher score of symptoms of depression and suicidality reflects some effectiveness of the system to direct patients to appropriate mental health care when screening signals opportunities for improvement of mental health. The observation that 39% of patients with suicidality were evaluated by a social worker reflects in part that many patients were already in mental health care, and in part, the need for maturation of our systems and strategies to better track and act on mental health screening using a comprehensive system-wide approach to address suicide risk. The current data set cannot discern the relative magnitude of these possibilities. A prior study found that patients who receive mental health care on the same day as a medical care visit are relatively more likely to initiate psychotherapy.^34^

## Conclusions

The findings that suicidality and symptoms of depression and anxiety are common among people seeking care for physical symptoms in specialty and non-specialty care, and that many, but not all were evaluated by an integrated social worker, demonstrates evidence for improvement in care designed to provide comprehensive, whole person care. Important factors associated with mental health as measured were younger age, English-speakers, and safety net insurance. The relationship between physical symptoms and mental health is bidirectional. People that seek care for physical symptoms often have distress about those symptoms. It is also true that people may feel more comfortable discussing stress and distress in terms of physical symptoms (somatic focus). Clinicians and clinical care units can structure their care to anticipate mental health priorities. Even in units with integrated social workers such as ours, there can be room for improvement in how mental health is screened, identified, discussed, further assessed, and addressed.

## Supplemental Material

sj-docx-1-css-10.1177_24705470231169106 - Supplemental material for Mental Health Among People Presenting for Care of Physical Symptoms: The Factors Associated with Suicidality and Symptoms of Depression and Anxiety are Similar Across Specialties Click here for additional data file. Supplemental material, sj-docx-1-css-10.1177_24705470231169106 for Mental Health Among People Presenting for Care of Physical Symptoms: The Factors Associated with Suicidality and Symptoms of Depression and Anxiety are Similar Across Specialties by Sina Ramtin, Kathleen Carberry, Maria Correa, David Ring, Carol Alter and Donna Shanor in Chronic Stress

sj-xlsx-2-css-10.1177_24705470231169106 - Supplemental material for Mental Health Among People Presenting for Care of Physical Symptoms: The Factors Associated with Suicidality and Symptoms of Depression and Anxiety are Similar Across Specialties Click here for additional data file. Supplemental material, sj-xlsx-2-css-10.1177_24705470231169106 for Mental Health Among People Presenting for Care of Physical Symptoms: The Factors Associated with Suicidality and Symptoms of Depression and Anxiety are Similar Across Specialties by Sina Ramtin, Kathleen Carberry, Maria Correa, David Ring, Carol Alter and Donna Shanor in Chronic Stress

sj-docx-3-css-10.1177_24705470231169106 - Supplemental material for Mental Health Among People Presenting for Care of Physical Symptoms: The Factors Associated with Suicidality and Symptoms of Depression and Anxiety are Similar Across Specialties Click here for additional data file. Supplemental material, sj-docx-3-css-10.1177_24705470231169106 for Mental Health Among People Presenting for Care of Physical Symptoms: The Factors Associated with Suicidality and Symptoms of Depression and Anxiety are Similar Across Specialties by Sina Ramtin, Kathleen Carberry, Maria Correa, David Ring, Carol Alter and Donna Shanor in Chronic Stress

sj-docx-4-css-10.1177_24705470231169106 - Supplemental material for Mental Health Among People Presenting for Care of Physical Symptoms: The Factors Associated with Suicidality and Symptoms of Depression and Anxiety are Similar Across Specialties Click here for additional data file. Supplemental material, sj-docx-4-css-10.1177_24705470231169106 for Mental Health Among People Presenting for Care of Physical Symptoms: The Factors Associated with Suicidality and Symptoms of Depression and Anxiety are Similar Across Specialties by Sina Ramtin, Kathleen Carberry, Maria Correa, David Ring, Carol Alter and Donna Shanor in Chronic Stress

sj-docx-5-css-10.1177_24705470231169106 - Supplemental material for Mental Health Among People Presenting for Care of Physical Symptoms: The Factors Associated with Suicidality and Symptoms of Depression and Anxiety are Similar Across Specialties Click here for additional data file. Supplemental material, sj-docx-5-css-10.1177_24705470231169106 for Mental Health Among People Presenting for Care of Physical Symptoms: The Factors Associated with Suicidality and Symptoms of Depression and Anxiety are Similar Across Specialties by Sina Ramtin, Kathleen Carberry, Maria Correa, David Ring, Carol Alter and Donna Shanor in Chronic Stress

sj-docx-6-css-10.1177_24705470231169106 - Supplemental material for Mental Health Among People Presenting for Care of Physical Symptoms: The Factors Associated with Suicidality and Symptoms of Depression and Anxiety are Similar Across Specialties Click here for additional data file. Supplemental material, sj-docx-6-css-10.1177_24705470231169106 for Mental Health Among People Presenting for Care of Physical Symptoms: The Factors Associated with Suicidality and Symptoms of Depression and Anxiety are Similar Across Specialties by Sina Ramtin, Kathleen Carberry, Maria Correa, David Ring, Carol Alter and Donna Shanor in Chronic Stress

sj-docx-7-css-10.1177_24705470231169106 - Supplemental material for Mental Health Among People Presenting for Care of Physical Symptoms: The Factors Associated with Suicidality and Symptoms of Depression and Anxiety are Similar Across Specialties Click here for additional data file. Supplemental material, sj-docx-7-css-10.1177_24705470231169106 for Mental Health Among People Presenting for Care of Physical Symptoms: The Factors Associated with Suicidality and Symptoms of Depression and Anxiety are Similar Across Specialties by Sina Ramtin, Kathleen Carberry, Maria Correa, David Ring, Carol Alter and Donna Shanor in Chronic Stress

sj-docx-8-css-10.1177_24705470231169106 - Supplemental material for Mental Health Among People Presenting for Care of Physical Symptoms: The Factors Associated with Suicidality and Symptoms of Depression and Anxiety are Similar Across Specialties Click here for additional data file. Supplemental material, sj-docx-8-css-10.1177_24705470231169106 for Mental Health Among People Presenting for Care of Physical Symptoms: The Factors Associated with Suicidality and Symptoms of Depression and Anxiety are Similar Across Specialties by Sina Ramtin, Kathleen Carberry, Maria Correa, David Ring, Carol Alter and Donna Shanor in Chronic Stress

sj-docx-9-css-10.1177_24705470231169106 - Supplemental material for Mental Health Among People Presenting for Care of Physical Symptoms: The Factors Associated with Suicidality and Symptoms of Depression and Anxiety are Similar Across Specialties Click here for additional data file. Supplemental material, sj-docx-9-css-10.1177_24705470231169106 for Mental Health Among People Presenting for Care of Physical Symptoms: The Factors Associated with Suicidality and Symptoms of Depression and Anxiety are Similar Across Specialties by Sina Ramtin, Kathleen Carberry, Maria Correa, David Ring, Carol Alter and Donna Shanor in Chronic Stress

## References

[1] Kroenke K Wu J Yu Z , et al. The patient health questionnaire anxiety and depression scale (PHQ-ADS): Initial validation in three clinical trials. Psychosom Med. 2016;78:716. 27187854 10.1097/PSY.0000000000000322 PMC4927366

[2] Mitchell AJ Vaze A Rao S . Clinical diagnosis of depression in primary care: A meta-analysis. Lancet. 2009;374:609–619. 19640579 10.1016/S0140-6736(09)60879-5

[3] Zuckerbrot RA Maxon L Pagar D , et al. Adolescent depression screening in primary care: Feasibility and acceptability. Pediatrics. 2007;119:101–108. 17200276 10.1542/peds.2005-2965

[4] Costantini L Pasquarella C Odone A , et al. Screening for depression in primary care with patient health questionnaire-9 (PHQ-9): A systematic review. J Affect Disord. 2021;279:473–483. 33126078 10.1016/j.jad.2020.09.131

[5] Mournet AM Bridge JA Ross A , et al. A Comparison of suicide attempt histories of pediatric and adult medical inpatients and implications for screening. Arch Suicide Res. Epub ahead of print 2021. DOI: 10.1080/13811118.2021.1931596 PMC11500749 34101537

[6] Teunis T Ramtin S Gwilym SE , et al. Unhelpful thoughts and distress regarding symptoms are associated with recovery from upper extremity fracture. Injury. Epub ahead of print 2023. DOI: 10.1016/j.injury.2023.02.035 36822916

[7] Gonzalez AI Ramtin S Ring D , et al. People have mixed reactions to both physiological and psychological explanations of disproportionate pain. Clin Orthop Relat Res. Epub ahead of print 8 March 2022. DOI: 10.1097/CORR.0000000000002163 PMC9191279 35258498

[8] Rohrback M Ramtin S Abdelaziz A , et al. Rotator cuff tendinopathy: magnitude of incapability is associated with greater symptoms of depression rather than pathology severity. J Shoulder Elbow Surg. Epub ahead of print April 2022. DOI: 10.1016/J.JSE.2022.03.007 35461981

[9] Chock MM Bommersbach TJ Geske JL , et al. Patterns of health care usage in the year before suicide: A population-based case-control study. Mayo Clin Proc. 2015;90:1475–1481. 26455886 10.1016/j.mayocp.2015.07.023 PMC4999076

[10] Uebelacker LA German NM Gaudiano BA , et al. Patient health questionnaire depression scale as a suicide screening instrument in depressed primary care patients: a cross-sectional study. Prim Care Companion CNS Disord. Epub ahead of print 2011. 13. DOI: 10.4088/PCC.10M01027 PMC3121214 21731830

[11] Al Salman A Shah R Thomas JE , et al. Symptoms of depression and catastrophic thinking attenuate the relationship of pain intensity and magnitude of incapability with fracture severity. J Psychosom Res. 2022;158:110915. 35483125 10.1016/j.jpsychores.2022.110915

[12] Sarwar F Crijns T Ramtin S , et al. Patient symptom exaggeration is associated with communication effectiveness and trust. PEC Innovation. 2022;1:100050. 10.1016/j.pecinn.2022.100050 PMC10194274 37213755

[13] Miner H Rijk L Thomas J , et al. Mental-Health phenotypes and patient-reported outcomes in upper-extremity illness. J Bone Joint Surg Am. 2021;103:1411–1416. 34357891 10.2106/JBJS.20.01945

[14] Na PJ Yaramala SR Kim JA , et al. The PHQ-9 item 9 based screening for suicide risk: A validation study of the patient health questionnaire (PHQ)-9 item 9 with the Columbia suicide severity rating scale (C-SSRS). J Affect Disord. 2018;232:34–40. 29477096 10.1016/j.jad.2018.02.045

[15] Bauer AM Chan YF Huang H , et al. Characteristics, management, and depression outcomes of primary care patients who endorse thoughts of death or suicide on the PHQ-9. J Gen Intern Med. 2013;28:363–369. 22936288 10.1007/s11606-012-2194-2 PMC3579977

[16] Valuck RJ Anderson HO Libby AM , et al. Enhancing electronic health record measurement of depression severity and suicide ideation: A distributed ambulatory research in therapeutics network (DARTNet) study. The Journal of the American Board of Family Medicine. 2012;25:582–593. 22956694 10.3122/jabfm.2012.05.110053

[17] Arias de la Torre J Ronaldson A Vilagut G , et al. Improving suicide surveillance systems through the use of the patient health questionnaire-9. J Affect Disord. 2021;293:71–72. 34174473 10.1016/j.jad.2021.06.008

[18] Viguera AC Milano N Laurel R , et al. Comparison of electronic screening for suicidal risk with the patient health questionnaire item 9 and the Columbia suicide severity rating scale in an outpatient psychiatric clinic. Psychosomatics. 2015;56:460–469. 26278339 10.1016/j.psym.2015.04.005

[19] Kroenke K Spitzer RL Williams JBW . The PHQ-9: Validity of a brief depression severity measure. J Gen Intern Med. 2001;16:606. 11556941 10.1046/j.1525-1497.2001.016009606.x PMC1495268

[20] Sapra A Bhandari P Sharma S , et al. Using Generalized Anxiety Disorder-2 (GAD-2) and GAD-7 in a Primary Care Setting. Cureus; 12. Epub ahead of print 21 May 2020. DOI: 10.7759/CUREUS.8224. PMC7306644 32582485

[21] Boersma EZ Kortlever JTP Loeb MD , et al. The Association Between Patient-Reported Outcome Measurement Scores and Preference for Specific Interventions. *J Patient Exp* 2020;7:1595–1601. DOI: 10.1177/2374373519897761 PMC7786652 33457619

[22] Chen H Cohen P Chen S . How Big is a Big Odds Ratio? Interpreting the Magnitudes of Odds Ratios in Epidemiological Studies. 2010;39:860–864. DOI: 101080/03610911003650383.

[23] Molleman J Janssen SJ Overbeek CL , et al. A threshold disability score corresponds with an estimated diagnosis of clinical depression in patients with upper extremity disease. Hand (N Y). 2015;10:168–172. 26034425 10.1007/s11552-014-9686-y PMC4447652

[24] Crijns TJ Brinkman N Ramtin S , et al. Are there distinct statistical groupings of mental health factors and pathophysiology severity among people with hip and knee osteoarthritis presenting for specialty care? Clin Orthop Relat Res. Epub ahead of print 24 November 2021. DOI: 10.1097/CORR.0000000000002052 PMC8747586 34817453

[25] Gonzalez AI Kortlever JTP Brown LE , et al. Can crafted communication strategies allow musculoskeletal specialists to address health within the biopsychosocial paradigm? Clin Orthop Relat Res. 2021;479:1217–1223. 33411452 10.1097/CORR.0000000000001635 PMC8133223

[26] Barsky AJ . The iatrogenic potential of the physician’s words. JAMA. 2017;318:2425–2426. 29090307 10.1001/jama.2017.16216

[27] Ring D . Pearls: Effective communication strategies for the biopsychosocial paradigm of musculoskeletal medicine. Clin Orthop Relat Res. 2020;478:2720–2721. 33136557 10.1097/CORR.0000000000001559 PMC7899385

[28] Califf RM Wong C Doraiswamy PM , et al. Importance of Social Determinants in Screening for Depression. DOI: 10.1007/s11606-021-06957-5. PMC9411454 34405346

[29] Schlax J Jünger C Beutel ME , et al. Income and education predict elevated depressive symptoms in the general population: results from the Gutenberg health study. DOI: 10.1186/s12889-019-6730-4. PMC6480596 31014301

[30] Bernstein DN Atkinson J Fear K , et al. Determining the generalizability of the PROMIS depression domain’s floor effect and completion time in patients undergoing orthopaedic surgery. Clin Orthop Relat Res. 2019;477:2215–2225. 31369439 10.1097/CORR.0000000000000782 PMC6999924

[31] Guattery JM Dardas AZ Kelly M , et al. Floor effect of PROMIS depression CAT associated with hasty completion in orthopaedic surgery patients. Clin Orthop Relat Res. 2018;476:696–703. 29419628 10.1007/s11999.0000000000000076 PMC6260068

[32] Badejo MA Ramtin S Rossano A , et al. Does adjusting for social desirability reduce ceiling effects and increase variation of patient-reported experience measures? J Patient Exp. 9. Epub ahead of print 1 February 2022. DOI: 10.1177/23743735221079144. PMC8829720 35155757

[33] Califf RM Wong C Doraiswamy PM , et al. Importance of social determinants in screening for depression. J Gen Intern Med. Epub ahead of print 2021. DOI: 10.1007/S11606-021-06957-5. PMC9411454 34405346

[34] Szymanski BR Bohnert KM Zivin K , et al. Integrated care: Treatment initiation following positive depression screens. J Gen Intern Med. 2013;28:346–352. 23150068 10.1007/s11606-012-2218-y PMC3579958

