# Supplementary material for: Mental Health Among People Presenting for Care of Physical Symptoms: The Factors Associated with Suicidality and Symptoms of Depression and Anxiety are Similar Across Specialties
Source: Chronic Stress (Thousand Oaks). 2023 Apr 18;7:24705470231169106. doi: 10.1177/24705470231169106 (PMC10123920; doi:10.1177/24705470231169106)
Supplement: sj-docx-1-css-10.1177_24705470231169106 - Supplemental material for Mental Health Among People Presenting for Care of Physical Symptoms: The Factors Associated with Suicidality and Symptoms of Depression and Anxiety are Similar Across Specialties [file sj-docx-1-css-10.1177_24705470231169106.docx]

| Appendix 1. Patient demographics | |
| --- | --- |
| **Appendix 1. Patient demographics** | **Value** |
| N | 13,211 |
| Age | 48 ± 17 |
| Gender |  |
| Man | 30% (3,963) |
| Woman | 70% (9,248) |
|  |  |
| Race |  |
| White | 68% (8,993) |
| Black or African American | 8.3% (1,096) |
| American Indian or Alaska Native | 0.94% (124) |
| Native Hawaiian or Other Pacific Island | 0.36% (47) |
| Asian | 4.7% (618) |
| Other Race | 0.46% (61) |
| Patient Declined | 17% (2,269) |
|  |  |
| Ethnicity |  |
| Hispanic or Latino/Spanish | 30% (3,996) |
| Not Hispanic or Latino | 59% (7,755) |
| Patient Declined | 11% (1457) |
|  |  |
| Language |  |
| English | 84% (11,097) |
| Spanish | 15% (1,941) |
| Others | 1.3% (173) |
|  |  |
| Insurance status |  |
| County insurance | 27% (3,569) |
| Medicaid | 4.7% (620) |
| Medicare | 16% (2,173) |
| Commercial | 48% (6,263) |
| Self-pay | 4.1% (542) |
|  |  |
| Integrated Practice Unit |  |
| Primary Care | 12% (1,633) |
| Medical Specialties | 9.3% (1,222) |
| Comprehensive Memory Center | 1.9% (251) |
| Women's Health | 25% (3,278) |
| Multiple Sclerosis & Neuroimmunology | 3.2% (420) |
| Musculoskeletal | 48% (6,294) |
| Comprehensive Pain Management | 0.86% (113) |
|  |  |
| Continuous variables as mean ± standard deviation; categorical variables as percentage (number). | |
