# Supplementary material for: Mental Health Among People Presenting for Care of Physical Symptoms: The Factors Associated with Suicidality and Symptoms of Depression and Anxiety are Similar Across Specialties
Source: Chronic Stress (Thousand Oaks). 2023 Apr 18;7:24705470231169106. doi: 10.1177/24705470231169106 (PMC10123920; doi:10.1177/24705470231169106)
Supplement: sj-docx-3-css-10.1177_24705470231169106 - Supplemental material for Mental Health Among People Presenting for Care of Physical Symptoms: The Factors Associated with Suicidality and Symptoms of Depression and Anxiety are Similar Across Specialties [file sj-docx-3-css-10.1177_24705470231169106.docx]

| Appendix 3. Logistic regression analysis of patient factors associated with the PHQ question 9 (suicidality) score of greater than 1 | | | |
| --- | --- | --- | --- |
| **Variables** | **Odd's ratio (95% Confidence Interval)** | **Standard Error** | ***P*-value** |
|  |  |  |  |
| Gender |  |  |  |
| Woman | *reference value* |  |  |
| Man | 1.25 (0.88 to 1.76) | 0.220 | 0.21 |
|  |  |  |  |
| Department |  |  |  |
| Primary Care | *reference value* |  |  |
| Medical Specialties | 1.09 (0.46 to 2.57) | 0.478 | 0.85 |
| Comprehensive Memory Center | 2.90 (1.09 to 7.72) | 1.448 | **0.03** |
| Women's Health | 0.45 (0.21 to 0.98) | 0.179 | **0.046** |
| Multiple Sclerosis & Neuroimmunology | 1.70 (0.67 to 4.30) | 0.805 | 0.26 |
| Musculoskeletal | 0.65 (0.32 to 1.33) | 0.237 | 0.24 |
| Comprehensive Pain Management | 0.32 (0.04 to 2.74) | 0.351 | 0.30 |
|  |  |  |  |
| Ethnicity |  |  |  |
| Non hispanic or Latino | *reference value* |  |  |
| Hispanic or Latino/Spanish | 1.04 (0.72 to 1.51) | 0.198 | 0.83 |
| Patient Declined | 1.51 (0.96 to 2.36) | 0.344 | 0.07 |
|  |  |  |  |
| Insurance status |  |  |  |
| County insurance | *reference value* |  |  |
| Medicaid | 1.07 (0.59 to 1.95) | 0.327 | 0.81 |
| Medicare | 0.50 (0.31 to 0.81) | 0.121 | **0.004** |
| Commercial | 0.44 (0.25 to 0.77) | 0.125 | **0.004** |
| Self-pay | 0.79 (0.37 to 1.71) | 0.311 | 0.55 |
|  |  |  |  |
| **Bold** indicates statistical significance, *P* < 0.05. PHQ-9= Patient Health Questionnaire, 9-item. | | | |
