# Supplementary material for: Mental Health Among People Presenting for Care of Physical Symptoms: The Factors Associated with Suicidality and Symptoms of Depression and Anxiety are Similar Across Specialties
Source: Chronic Stress (Thousand Oaks). 2023 Apr 18;7:24705470231169106. doi: 10.1177/24705470231169106 (PMC10123920; doi:10.1177/24705470231169106)
Supplement: sj-docx-4-css-10.1177_24705470231169106 - Supplemental material for Mental Health Among People Presenting for Care of Physical Symptoms: The Factors Associated with Suicidality and Symptoms of Depression and Anxiety are Similar Across Specialties [file sj-docx-4-css-10.1177_24705470231169106.docx]

| Appendix 4. Logistic regression analysis of patient factors associated with PHQ score greater than 0 | | | |
| --- | --- | --- | --- |
| **Variables** | **Odd's ratio (95% Confidence Interval)** | **Standard Error** | ***P-value*** |
|  |  |  |  |
| Gender |  |  |  |
| Woman | *reference value* |  |  |
| Man | 0.78 (0.72 to 0.85) | 0.034 | **<0.001** |
|  |  |  |  |
| Department |  |  |  |
| Primary Care | *reference value* |  |  |
| Medical Specialties | 1.29 (1.10 to 1.50) | 0.101 | **0.001** |
| Comprehensive Memory Center | 1.75 (1.30 to 2.35) | 0.263 | **<0.001** |
| Women's Health | 1.69 (1.48 to 1.93) | 0.113 | **<0.001** |
| Multiple Sclerosis & Neuroimmunology | 2.33 (1.87 to 2.91) | 0.263 | **<0.001** |
| Musculoskeletal | 1.60 (1.41 to 1.81) | 0.099 | **<0.001** |
| Comprehensive Pain Management | 2.84 (1.83 to 4.39) | 0.631 | **<0.001** |
|  |  |  |  |
| Language |  |  |  |
| Spanish | *reference value* |  |  |
| English | 1.67 (1.48 to 1.89) | 0.106 | **<0.001** |
| Other | 1.76 (1.25 to 2.46) | 0.304 | 0.77 |
|  |  |  |  |
| Insurance status |  |  |  |
| County insurance | *reference value* |  |  |
| Medicaid | 1.04 (0.85 to 1.27) | 0.106 | 0.68 |
| Medicare | 0.58 (0.50 to 0.67) | 0.042 | **<0.001** |
| Commercial | 0.43 (0.38 to 0.48) | 0.024 | **<0.001** |
| Self-pay | 0.58 (0.48 to 0.72) | 0.061 | **<0.001** |
|  |  |  |  |
| Age | 0.994 (0.991 to 0.997) | 0.001 | **<0.001** |
|  |  |  |  |
| **Bold indicates statistical significance, P < 0.05. Race and ethnicity were dropped because of the collinearity with language. PHQ-9= Patient Health Questionnaire** | | | |
