# Supplementary material for: Mental Health Among People Presenting for Care of Physical Symptoms: The Factors Associated with Suicidality and Symptoms of Depression and Anxiety are Similar Across Specialties
Source: Chronic Stress (Thousand Oaks). 2023 Apr 18;7:24705470231169106. doi: 10.1177/24705470231169106 (PMC10123920; doi:10.1177/24705470231169106)
Supplement: sj-docx-5-css-10.1177_24705470231169106 - Supplemental material for Mental Health Among People Presenting for Care of Physical Symptoms: The Factors Associated with Suicidality and Symptoms of Depression and Anxiety are Similar Across Specialties [file sj-docx-5-css-10.1177_24705470231169106.docx]

| Appendix 5. Logistic regression analysis of patient factors associated with the PHQ score of 3 or greater | | | |
| --- | --- | --- | --- |
| **Variables** | **Odd's ratio (95% Confidence Interval)** | **Standard Error** | ***P*-value** |
|  |  |  |  |
| Department |  |  |  |
| Primary Care | *reference value* |  |  |
| Medical Specialties | 1.50 (1.23 to 1.82) | 0.151 | **0.004** |
| Comprehensive Memory Center | 1.66 (1.17 to 2.34) | 0.291 | **0.001** |
| Women's Health | 2.02 (1.72 to 2.39) | 0.170 | **<0.001** |
| Multiple Sclerosis & Neuroimmunology | 2.28 (1.76 to 2.95) | 0.301 | **<0.001** |
| Musculoskeletal | 2.11 (1.80 to 2.47) | 0.170 | **<0.001** |
| Comprehensive Pain Management | 3.26 (2.16 to 4.92) | 0.685 | **<0.001** |
|  |  |  |  |
| Language |  |  |  |
| Spanish | *reference value* |  |  |
| English | 1.42 (1.25 to 1.61) | 0.090 | **<0.001** |
| Other | 1.64 (1.18 to 2.28) | 0.274 | **0.003** |
|  |  |  |  |
| Insurance status |  |  |  |
| County insurance | *reference value* |  |  |
| Medicaid | 1.16 (0.96 to 1.39) | 0.109 | 0.12 |
| Medicare | 0.60 (0.52 to 0.70) | 0.044 | **<0.001** |
| Commercial | 0.40 (0.36 to 0.45) | 0.023 | **<0.001** |
| Self-pay | 0.53 (0.43 to 0.67) | 0.061 | **<0.001** |
|  |  |  |  |
| Age | 1.001 (0.998 to 1.004) | 0.481 | 0.48 |
|  |  |  |  |
| **Bold** indicates statistical significance, *P* < 0.05. Race and ethnicity were dropped because of the collinearity with language. GAD = General Anxiety Disorders | | | |
