# Supplementary material for: Mental Health Among People Presenting for Care of Physical Symptoms: The Factors Associated with Suicidality and Symptoms of Depression and Anxiety are Similar Across Specialties
Source: Chronic Stress (Thousand Oaks). 2023 Apr 18;7:24705470231169106. doi: 10.1177/24705470231169106 (PMC10123920; doi:10.1177/24705470231169106)
Supplement: sj-docx-6-css-10.1177_24705470231169106 - Supplemental material for Mental Health Among People Presenting for Care of Physical Symptoms: The Factors Associated with Suicidality and Symptoms of Depression and Anxiety are Similar Across Specialties [file sj-docx-6-css-10.1177_24705470231169106.docx]

| Appendix 6. Logistic regression analysis of patient factors associated with GAD score of 3 or greater | | | |
| --- | --- | --- | --- |
| **Variables** | **Odd's ratio (95% Confidence Interval)** | **Standard Error** | ***P*-value** |
|  |  |  |  |
| Gender |  |  |  |
| Woman | *reference value* |  |  |
| Man | 0.73 (0.66 to 0.81) | 0.037 | **<0.001** |
|  |  |  |  |
| Department |  |  |  |
| Primary Care | *reference value* |  |  |
| Medical Specialties | 1.22 (1.01 to 1.48) | 0.120 | **0.040** |
| Comprehensive Memory Center | 1.80 (1.28 to 2.54) | 0.315 | **0.001** |
| Women's Health | 1.48 (1.27 to 1.74) | 0.119 | **<0.001** |
| Multiple Sclerosis & Neuroimmunology | 1.81 (1.41 to 2.32) | 0.231 | **<0.001** |
| Musculoskeletal | 1.45 (1.25 to 1.69) | 0.111 | **<0.001** |
| Comprehensive Pain Management | 2.93 (1.95 to 4.41) | 0.610 | **<0.001** |
|  |  |  |  |
| Language |  |  |  |
| Spanish | *reference value* |  |  |
| English | 1.89 (1.65 to 2.16) | 0.131 | **<0.001** |
| Other | 1.72 (1.21 to 2.44) | 0.307 | **0.002** |
|  |  |  |  |
| Insurance status |  |  |  |
| County insurance | *reference value* |  |  |
| Medicaid | 1.04 (0.86 to 1.27) | 0.102 | 0.65 |
| Medicare | 0.69 (0.59 to 0.80) | 0.054 | **<0.001** |
| Commercial | 0.51 (0.45 to 0.57) | 0.031 | **<0.001** |
| Self-pay | 0.66 (0.53 to 0.83) | 0.076 | **<0.001** |
|  |  |  |  |
| Age | 0.986 (0.983 to 0.989) | 0.002 | **<0.001** |
|  |  |  |  |
| **Bold** indicates statistical significance, *P* < 0.05. Race was dropped because of the collinearity with language. GAD = General Anxiety Disorders | | | |
