# Supplementary material for: Mental Health Among People Presenting for Care of Physical Symptoms: The Factors Associated with Suicidality and Symptoms of Depression and Anxiety are Similar Across Specialties
Source: Chronic Stress (Thousand Oaks). 2023 Apr 18;7:24705470231169106. doi: 10.1177/24705470231169106 (PMC10123920; doi:10.1177/24705470231169106)
Supplement: sj-docx-7-css-10.1177_24705470231169106 - Supplemental material for Mental Health Among People Presenting for Care of Physical Symptoms: The Factors Associated with Suicidality and Symptoms of Depression and Anxiety are Similar Across Specialties [file sj-docx-7-css-10.1177_24705470231169106.docx]

| Appendix 7. Logistic regression analysis of patient factors associated with PHQ score or GAD score of 3 or greater | | | |
| --- | --- | --- | --- |
| **Variables** | **Odd's ratio (95% (Confidence Interval)** | **Standard Error** | ***P*-value** |
|  |  |  |  |
| Gender |  |  |  |
| Woman | *reference value* |  |  |
| Man | 0.81 (0.74 to 0.88) | 0.036 | **<0.001** |
|  |  |  |  |
| Department |  |  |  |
| Primary Care | *reference value* |  |  |
| Medical Specialties | 1.33 (1.12 to 1.58) | 0.116 | **0.001** |
| Comprehensive Memory Center | 1.77 (1.30 to 2.40) | 0.278 | **<0.001** |
| Women's Health | 1.84 (1.59 to 2.12) | 0.134 | **<0.001** |
| Multiple Sclerosis & Neuroimmunology | 2.09 (1.66 to 2.63) | 0.245 | **<0.001** |
| Musculoskeletal | 1.97 (1.72 to 2.25) | 0.135 | **<0.001** |
| Comprehensive Pain Management | 3.41 (2.28 to 5.10) | 0.700 | **<0.001** |
|  |  |  |  |
| Language |  |  |  |
| Spanish | *reference value* |  |  |
| English | 1.46 (1.29 to 1.64) | 0.089 | **<0.001** |
| Other | 1.48 (1.07 to 2.03) | 0.241 | **0.02** |
|  |  |  |  |
| Insurance status |  |  |  |
| County insurance | *reference value* |  |  |
| Medicaid | 1.13 (0.95 to 1.36) | 0.106 | 0.17 |
| Medicare | 0.65 (0.57 to 0.75) | 0.046 | **<0.001** |
| Commercial | 0.47 (0.42 to 0.53) | 0.042 | **<0.001** |
| Self-pay | 0.62 (0.51 to 0.76) | 0.065 | **<0.001** |
|  |  |  |  |
| Age | 0.993 (0.991 to 0.996) | 0.001 | **<0.001** |
|  |  |  |  |
| **Bold** indicates statistical significance, *P* < 0.05. Race and ethinicity were dropped because of the collinearity with language. PHQ-9= Patient Health Questionnaire, 9-item. GAD = General Anxiety Disorders | | | |
