# Supplementary material for: Mental Health Among People Presenting for Care of Physical Symptoms: The Factors Associated with Suicidality and Symptoms of Depression and Anxiety are Similar Across Specialties
Source: Chronic Stress (Thousand Oaks). 2023 Apr 18;7:24705470231169106. doi: 10.1177/24705470231169106 (PMC10123920; doi:10.1177/24705470231169106)
Supplement: sj-docx-8-css-10.1177_24705470231169106 - Supplemental material for Mental Health Among People Presenting for Care of Physical Symptoms: The Factors Associated with Suicidality and Symptoms of Depression and Anxiety are Similar Across Specialties [file sj-docx-8-css-10.1177_24705470231169106.docx]

| Appendix 8. Logistic regression analysis of patient factors associated with the PHQ score of 10 or greater | | | |
| --- | --- | --- | --- |
| **Variables** | **Odd's ratio (95% Confidence Interval)** | **Standard Error** | ***P*-value** |
|  |  |  |  |
| Gender |  |  |  |
| Woman | *reference value* |  |  |
| Man | 0.81 (0.73 to 0.90) | 0.044 | **<0.001** |
|  |  |  |  |
| Department |  |  |  |
| Primary Care | *reference value* |  |  |
| Medical Specialties | 1.83 (1.43 to 2.34) | 0.231 | **<0.001** |
| Comprehensive Memory Center | 2.45 (1.65 to 3.65) | 0.496 | **<0.001** |
| Women's Health | 2.45 (1.98 to 3.02) | 0.264 | **<0.001** |
| Multiple Sclerosis & Neuroimmunology | 2.82 (2.08 to 3.84) | 0.442 | **<0.001** |
| Musculoskeletal | 2.72 (2.23 to 3.33) | 0.281 | **<0.001** |
| Comprehensive Pain Management | 4.09 (2.61 to 6.39) | 0.933 | **<0.001** |
|  |  |  |  |
| Language |  |  |  |
| Spanish | *reference value* |  |  |
| English | 1.67 (1.45 to 1.93) | 0.120 | **<0.001** |
| Other | 1.54 (1.07 to 2.22) | 0.286 | **0.019** |
|  |  |  |  |
| Insurance status |  |  |  |
| County insurance | *reference value* |  |  |
| Medicaid | 1.16 (0.96 to 1.41) | 0.116 | 0.13 |
| Medicare | 0.40 (0.35 to 0.45) | 0.026 | **<0.001** |
| Commercial | 0.60 (0.51 to 0.70) | 0.049 | **<0.001** |
| Self-pay | 0.50 (0.38 to 0.64) | 0.066 | **<0.001** |
|  |  |  |  |
| Age | 1.00 (0.997 to 1.003) | 0.002 | 0.73 |
|  |  |  |  |
| **Bold** indicates statistical significance, *P* < 0.05. Race and ethnicity were dropped because of the collinearity with language. PHQ-9= Patient Health Questionnaire, 9-item. | | | |
