# Supplementary material for: Mental Health Among People Presenting for Care of Physical Symptoms: The Factors Associated with Suicidality and Symptoms of Depression and Anxiety are Similar Across Specialties
Source: Chronic Stress (Thousand Oaks). 2023 Apr 18;7:24705470231169106. doi: 10.1177/24705470231169106 (PMC10123920; doi:10.1177/24705470231169106)
Supplement: sj-docx-9-css-10.1177_24705470231169106 - Supplemental material for Mental Health Among People Presenting for Care of Physical Symptoms: The Factors Associated with Suicidality and Symptoms of Depression and Anxiety are Similar Across Specialties [file sj-docx-9-css-10.1177_24705470231169106.docx]

| Appendix 9. Negative Binomial regression analysis of patient factors associated with GAD score | | | |
| --- | --- | --- | --- |
| **Variables** | **Regression Coefficient  (95% Confidence Interval)** | **Standard Error** | ***P*-value** |
|  |  |  |  |
| Gender |  |  |  |
| Woman | *reference value* |  |  |
| Man | -0.25 (-0.32 to -0.17) | 0.038 | **<0.001** |
|  |  |  |  |
| Department |  |  |  |
| Primary Care | *reference value* |  |  |
| Medical Specialties | 0.17 (0.03 to 0.30) | 0.070 | **0.017** |
| Comprehensive Memory Center | 0.78 (0.53 to 1.04) | 0.13 | **<0.001** |
| Women's Health | 0.37 (0.26 to 0.48) | 0.058 | **<0.001** |
| Multiple Sclerosis & Neuroimmunology | 0.62 (0.43 to 0.81) | 0.098 | **<0.001** |
| Musculoskeletal | 0.22 (0.11 to 0.33) | 0.055 | **<0.001** |
| Comprehensive Pain Management | 0.75 (0.41 to 1.09) | 0.18 | **<0.001** |
|  |  |  |  |
| Language |  |  |  |
| Spanish | *reference value* |  |  |
| English | 0.55 (0.45 to 0.65) | 0.052 | **<0.001** |
| Other | 0.52 (0.23 to 0.80) | 0.14 | **<0.001** |
|  |  |  |  |
| Insurance status |  |  |  |
| County insurance | *reference value* |  |  |
| Medicaid | -0.03 (-0.18 to 0.13) | 0.081 | 0.76 |
| Medicare | -0.68 (-0.78 to -0.59) | 0.048 | **<0.001** |
| Commercial | -0.39 (-0.51 to -0.28) | 0.059 | **<0.001** |
| Self-pay | -0.49 (-0.66 to -0.32) | 0.089 | **<0.001** |
|  |  |  |  |
| Age | -0.01 (-0.02 to -0.01) | 0.0012 | **<0.001** |
|  |  |  |  |
| **Bold** indicates statistical significance, *P* < 0.05. Race and ethnicity were dropped because of the collinearity with language. PHQ-9= Patient Health Questionnaire, 9-item. GAD = General Anxiety Disorders | | | |
